# In Situ Formation of Mechanically Interlocked Heterointerfaces with Ultrahigh Bonding Strength for Mg‐Based Multilayered Sheets

**DOI:** 10.1002/advs.202508319

**Published:** 2025-06-30

**Authors:** Zhilei Yu, Wenbo Luo, Wei Xue, Rongqin Xu, Xiuzhu Han, Zhiyong Xue, Wanshun Zhang, Hongyang Zhao, Xiaoming Cui

**Affiliations:** ^1^ Institute for Advanced Materials North China Electric Power University Beijing 102208 China; ^2^ Beijing Advanced Innovation Center for Materials Genome Engineering University of Science and Technology Beijing Beijing 100083 China; ^3^ School of Materials and Metallurgy University of Science and Technology Liaoning Liaoning 114051 China; ^4^ School of Materials Science and Engineering Inner Mongolia University of Technology Hohhot 010051 China

**Keywords:** heterointerfaces, laminated metal composites, mechanical interlock, Mg‐based alloys, molecular dynamics

## Abstract

Tailoring heterointerfaces is crucial for developing novel laminated metal composites (LMCs) with synergistic multifunctional structural properties. This study pioneers a novel approach to fabricating Mg‐Ta incompatible LMCs via crystal orientation regulation, achieving in situ formed serrated mechanically interlocked interfaces with exceptional bonding strength (80.5 MPa) through hot‐rolling. It also exhibits exceptional tensile properties with an ultimate tensile strength of 340–395 MPa, a yield strength of 301–363 MPa, and an elongation of 5–10.2%. Systematic multiscale investigations combining molecular dynamics (MD) simulations and density functional theory (DFT) calculations reveal a hierarchical interfacial architecture comprising dual heterointerfaces zone (hexagonal close‐packed*/face center cubic* Mg/Cu and face centered cubic*/body‐centered cubic*Cu/Ta), with strain‐modulated micro‐serrations (width: 1.6–5.5 µm; depth: 6–15 µm) morphology, along with atomic interdiffusion zones (1.1–3.1 µm). A quantifiable inverse correlation is identified between serration dimensions and interfacial strength, with reduced width and depth directly enhancing bond integrity. Furthermore, the heterointerface evolution mechanism is decoupled into three thermomechanical stages: 1) crystallographic orientation optimization via dissimilar atomic contact, 2) strain‐driven micro‐sawtooth generation through dislocation slip competition, and 3) geometric interlocking completion via interface self‐assembly. This work establishes a universal paradigm for designing multifunctional structural materials through strain‐engineered heterointerface manipulation, rather than just applying several specific deformations processes.

## Graphical abstract

Crystal‐orientation controlled hot‐rolling strategy creates Mg‐Ta immiscible composites featuring mechanically interlocked in situ formed interfaces (80.5 MPa bond strength) and excellent tensile properties (340–395 MPa UTS). Systematic multiscale investigations reveal a serration‐size‐dependent bonding mechanism and a three‐stage interfacial evolution process, offering a universal paradigm for designing high‐performance immiscible metal composites through strain‐engineered heterointerface manipulation.

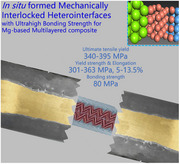

## 1 Introduction

With the rapid advancement of deep‐space exploration, the spacecraft satellite faces intense radiation exposure in extraterrestrial environment. Consequently, and multifunctional‐structural material containing ultrahigh‐performance‐combining essential attributes such as lightweight, high strength, radiation resistance, along with superior rigidity, have become indispensable for advanced spacecraft applications.^[^ ^1^, ^2^, ^3^ ^]^ Magnesium (Mg) alloys, as the most lightweight metallic structural metal, have demonstrated significant application in space station and transportation fields (e.g., Electric Vehicle, EV).^[^ ^4^, ^5^ ^]^ However, the intrinsic trade‐offs relationship, such as strength *vs*. plasticity, low density vs. radiation shielding property, as well as high specific strength vs. poor corrosion, severely restricts their further application in the above fields.^[^ ^6^, ^7^, ^8^, ^9^, ^10^, ^11^ ^]^ Therefore, it is urgently needed to explore a new approach to break through the aforementioned dilemma and then motivate its widespread application.

Laminated metal composites (LMCs) combine different kinds of materials exhibiting novel high‐performances: lightweight Mg/Al,^[^ ^12^ ^]^ and high specific‐strength and outstanding corrosion resistance of Mg/Ti,^[^ ^13^ ^]^ Al/Ta^[^ ^14^ ^]^ LMCs, and integrated great electromagnetic shielding with low density Al/Steel,^[^ ^15^ ^]^ Mg/Cu,^[^ ^16^ ^]^ Ti/Al/Mg,^[^ ^17^ ^]^ Mg/Al/Ta.^[^ ^18^ ^]^ Many researchers investigated the relationship between properties and the prepared processes of LMCs. Frank et al. found that Al/Steel laminated composites exhibit excellent monotonic mechanical properties, cyclic mechanical properties, and fatigue properties.^[^ ^15^ ^]^ Yao et al. prepared multilayered Cu mesh/ZK61 Mg foil composites with great thermal conductivity and lightweight mechanical properties through the diffusion bonding method.^[^ ^16^ ^]^ Feng et al. prepared a Ti/Mg/Ti composite, which exhibits better mechanical properties, including strength and ductility compared with AZ31 alloy alone.^[^ ^13^ ^]^

Although many Mg‐based LMCs were prepared, their interface bonding strength was still relatively low. For example, the bonding strength of Mg/Al LMCs^[^ ^12^ ^]^ was about ∼30 MPa, and the highest bonding strength of 64 MPa to date has been achieved for AZ31B/5052 composite plates,^[^ ^19^ ^]^ which were prepared by a unique process‐the corrugated rolling technique. Their relatively low interfacial bonding strength remains a major issue restricting their application.

Besides, the heterogeneous interface regulation mechanism of Mg‐based LMCs remains unclear, especially under the coupling effect of multiple factors: temperature, pressure, strain, texture, and so on. And these matrix metals exhibit great disparities in physical property, like crystal structure, melting points (*T_m_*), coefficients of thermal expansion (CTE), thermal conductivity (*α*), specific heat capacity (*c*), and even atomic radius (*r_0_*), mechanical strength and plasticity (*σ*, *e*), which significantly increase the complexity of precisely designing and regulating the interface. Moreover, the crystalline lattice of different metals also shows huge difference, it is well‐known that there is mainly 3 kinds of crystalline lattice are body‐centered cubic (*bcc*), face‐centered cubic (*fcc*), and hexagonal close‐packed (*hcp*) for most metals. The predominant slipping systems are different each other, such as {110}<111˃ for bcc, {111}<110˃ for *fcc*, and {0001}<11‐20˃ for *hcp*.^[^ ^20^, ^21^, ^22^ ^]^ Nevertheless, the heterogeneous crystalline structure provides a new approach to designing and preparing novel interfaces, based on the precise slipping orientation relationship. Zhang W.L. et al. designed and fabricated an Al/Ta bilayer hetero‐crystalline interface structure, which is a typical *fcc/bc*c interface.^[^ ^14^ ^]^ Meanwhile, Mg/Al and Mg/Cu exhibit typical *hcp/fcc* bilayer interfaces.^[^ ^16^, ^23^ ^]^ However, those metals with *hcp* and *bcc* structure (like Ta, W, and V), have rarely been studied so far.

In this work, we propose a novel approach for hetero‐interfaces tailoring by precise design of dissimilar crystalline orientation relationship, and then successfully prepared the Mg/Cu/Ta LMCs with *hcp/fcc/bcc* structure by hot‐rolling process, achieving in situ interlocked interface with high bonding strength. The interface's microstructure evolution and forming mechanism were also investigated in detail. Mg/Cu/Ta composites show outstanding properties such as light‐weight, high strength, and radiation resistance, and have demonstrated attractive potential in deep‐space exploration (e.g., solar system and Jupiter's exploration), where most of the traditional Mg alloys cannot meet all these requirements simultaneously.

## 2 Results

### 2.1 Interface Design and Calculation for Heterogeneous *hcp/fcc/bcc* Structure

**Figure 1:**
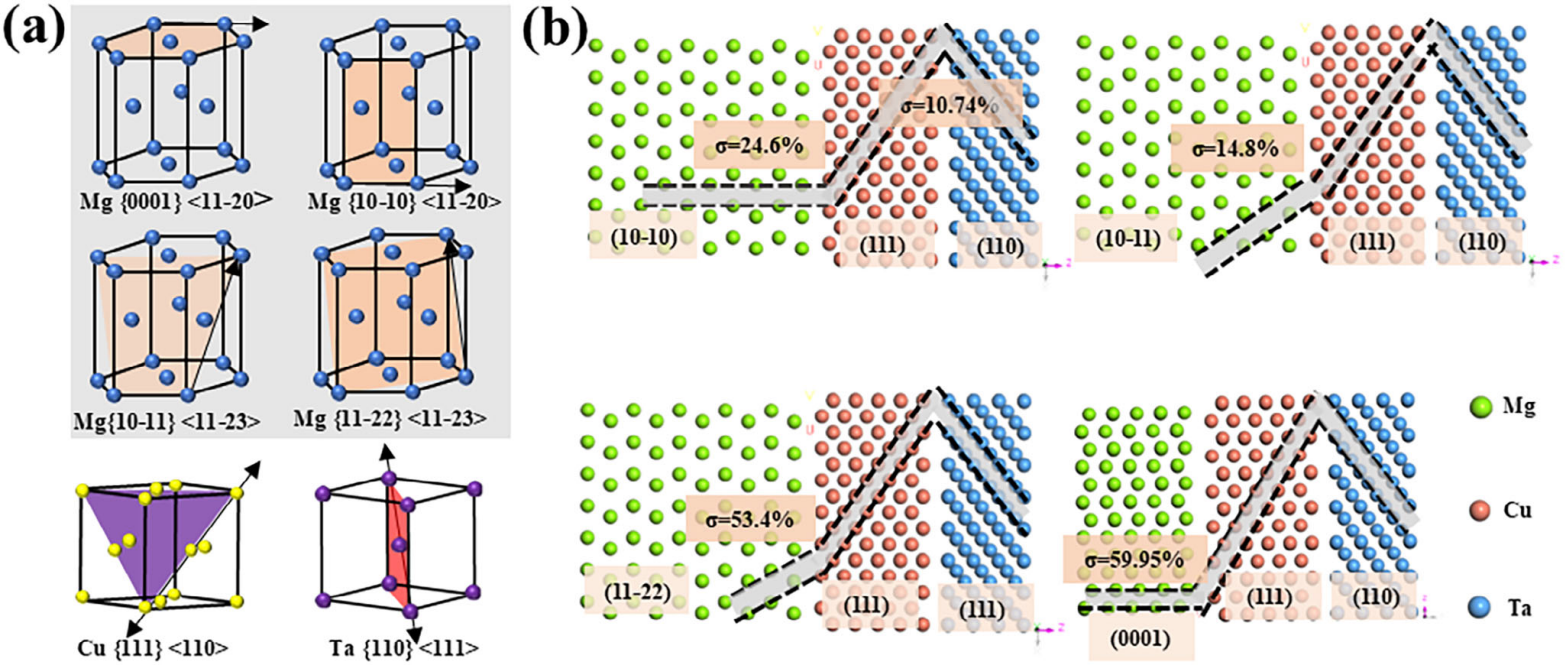
Heterogeneous *hcp/fcc/bcc* interfaces atom structure, a) slipping systems of Mg, Cu, and Ta metal, and b) different interface and corresponding interface mismatch.

**Figure** **1**a shows the main crystalline planes for Mg, Cu, and Ta metal, respectively. Basing these single plane, the designed interface combinations are displayed in Figure 1b. Different kind of planes contacted each other, and then occurring the plane mismatch, which was a key factor to evaluate atoms stacking mismatch, their values were calculated by interplanar spacing on the matched crystal planes, as presented in Equation (1):^[^ ^24^ ^]^

(1) $$\sigma =\frac{\left|\Delta \alpha_{0}\right|}{\alpha_{0}}\times 100\%$$

where *∆α_0_* represents the difference in interplanar spacing between two planes. *a_0_* represents the interplanar spacing of the matrix on a specific crystal plane, and *σ* is the interplanar spacing mismatch. Figure 1b displays the interface's atomic stacking structure. The crystallin plane mismatch values are listed in **Table** **1**. It can be seen that (10‐12)_Mg_/(111)_Cu_/(110)_Ta_ interfaces show the lowest mismatch, 14.8%/10.74%, meaning the appropriate lattice matching with a certain coherent orientation relation.^[^ ^24^ ^]^ The prismatic plane type, (10‐10)_Mg_/(111)_Cu_/(110)_Ta_, shows a slightly bigger mismatch, 24.6%/10.74%. While the pyramidal II type, (11‐22)_Mg_/(111)_Cu_/(110)_Ta_, exhibits great mismatch (53.4%) with a non‐coherent relation, too. And the *σ* value reaches up to 59.95%/10.74% for (0001)_Mg_/(111)_Cu_/(110)_Ta_ interface, it is because of the extremely large interplanar spacing of basal (0001)_Mg_, as well as the failure to consider the mechanical compatibility related to orientation‐dependent, particularly slip system activation thresholds under multiaxial loading conditions.

**Table 1:** The plane mismatch values for the Mg‐Cu‐Ta interface.

NO.	interface	*d_Mg_* [Å]	*d_Ta_* [Å]	d_Cu_ [Å]	Δ*a*_0_	*σ*
1	Mg (0001)/Ta (110)	5.211	2.338	–	2.873	122.88%
2	Mg (10‐10)/Ta (110)	2.77	2.338	–	0.432	18.48%
3	Mg (10‐11)/Ta (110)	2.45	2.338	–	0.112	4.79%
4	Mg (10‐12)/Ta (110)	1.9	2.338	–	0.438	18.73%
5	Mg (11‐22)/Ta (110)	1.36	2.338	–	0.978	41.83%
6	Ta (110)/Cu (111)	–	2.338	2.087	0.251	10.74%
7	Ta (110)/Cu (200)	–	2.338	1.808	0.53	22.67%
8	Ta (110)/Cu (110)	–	2.338	2.556	0.218	9.30%
9	Ta (111)/Cu (110)	–	1.91	1.278	0.632	33%
10	Ta (211)/Cu (111)	–	1.35	2.087	0.737	35.31%
11	Ta (211)/Cu (200)	–	1.35	1.808	0.458	25.33%
12	Ta (211)/Cu (110)	–	1.35	1.278	0.072	5.30%
13	Ta (321)/Cu (111)	–	0.884	2.087	1.203	57.64%
14	Ta (321)/Cu (200)	–	0.884	1.808	0.924	51.11%
15	Ta (321)/Cu (110)	–	0.884	1.278	0.394	30.83%
16	Mg (0001)/Cu (111)	5.211	–	2.087	3.124	59.95%
17	Mg (10‐10)/Cu (111)	2.77	–	2.087	0.683	24.60%
18	Mg (10‐11)/Cu (111)	2.45	–	2.087	0.363	14.80%
19	Mg (10‐12)/Cu (111)	1.9	–	2.087	0.187	9.80%
20	Mg (11‐22)/Cu (111)	1.36	–	2.087	0.727	53.40%
21	Mg (0001)/Cu (110)	5.211	–	2.556	2.655	50.95%
22	Mg (10‐10)/Cu (110)	2.77	–	2.556	0.683	7.73%
23	Mg (10‐11)/Cu (110)	2.45	–	2.556	0.363	4.33%
24	Mg (10‐12)/Cu (110)	1.9	–	2.556	0.656	34.53%
25	Mg (11‐22)/Cu (110)	1.36	–	2.556	0.727	87.94%

**Figure 2:**
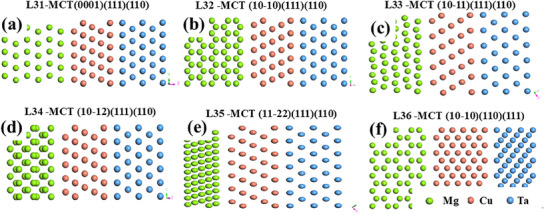
The designed Mg/Cu/Ta (MCT) interfaces with different atoms stacking planes, a) (0001)_Mg_/(111)_Cu_/(110)_Ta_, b) (10‐10)_Mg_/(111)_Cu_/(110)_Ta_, c) (10‐11)_Mg_/(111)_Cu_/(110)_Ta_, d) (10‐12)_Mg_/(111)_Cu_/(110)_Ta_, e) (11‐22)_Mg_/(111)_Cu_/(110)_Ta_, and f) (10‐10)_Mg_/(110)_Cu_/(111)_Ta_.

**Figure** **2** presents the 6 most probable interface models (L31‐36) identified from the DFT calculation. Following model construction and geometric optimization, the interfacial separation work (*W_sep_*), a key mechanical‐factor for evaluating the interface structure stability,^[^ ^25^ ^]^ was also calculated. This parameter defines the minimum energy required to cleave an interface in practice,^[^ ^26^ ^]^ as quantified by Equation (2). The calculated *W_sep_* values for all models are listed in **Table** **2**.

(2) $$W_{\mathrm{sep}}=\frac{E_{1}^{\mathrm{tot}}+E_{2}^{\mathrm{tot}}+E_{3}^{\mathrm{tot}}{-E}_{123}^{\mathrm{tot}}}{A}$$

where $E_{i}^{tot}$ and $E_{123}^{tot}$ represent the total energy of the constituent layer and interface (*eV*), respectively. A denotes the interface area (*Å^2^*). The results show that heterogeneous (*hcp/fcc/bcc*) configurations exhibit strong bonding, the values of *W_sep_* are predominantly distributed between 5.554 and 8.166 J m^−2^. And the (0001)_Mg_/(111)_Cu_/(110)_Ta_ heterostructure even achieves exceptional adhesion, 34.947 J m^−2^. These values are significantly greater than those of binary crystalline structure (0.304–5.135 J m^−2^) and homogeneous single structures (1.137–1.774 J m^−2^). It suggests that the heterogeneous interfaces possess promising static bonding behavior, although its interplanar spacing mismatch (*σ*) was greater than 20%.

**Table 2:** The interface energy of different *hcp/fcc/bcc* systems (J m^−2^).

Interface	Mg layer $E_{1}^{tot}/\left[eV\right]$	Ta layer $E_{2}^{tot}/\left[eV\right]$	Cu layer $E_{3}^{tot}/\left[eV\right]$	Interfacial cell $E_{123}^{tot}/\left[eV\right]$	Interface aera/[*Å^2^*]	W [*J m* ^−^ *^2^*]
**Single‐structure**						
Mg 0001	−16901.623	‐ ‐	‐ ‐	−33803.911	9.373	1.137
Mg 10‐11	−16903.450	‐ ‐	‐ ‐	−33809.001	18.970	1.774
**Binary‐structure**						
Cu 111 Ta 110	‐ ‐	−50599.364	−10072.879	−60678.090	18.240	5.135
Mg 0001 Cu 111	−20281.000	‐ ‐	−10082.41	−30364.189	16.000	0.779
Mg 10‐10 Cu 111	−20277.980	‐ ‐	−10079.78	−30359.497	29.210	0.953
Mg 10‐11 Cu 111	−10140.900	‐ ‐	−8391.111	−18533.455	15.919	1.454
Mg 10‐12 Cu 111	−20282.724	‐ ‐	−10066.634	−30354.500	45.260	1.820
Mg 0001 Ta 110	−20278.735	−50596.151	‐ ‐	−70875.277	20.64	0.304
**Multiple‐structure**						
MCT 0001‐111‐110	−16900.978	−84307.025	−33570.211	−134819.009	18.700	34.947
MCT 10‐10‐111‐110	−16902.230	−84307.025	−33570.211	−134787.720	23.800	5.554
MCT 10‐11‐111‐110	−16903.752	−84307.025	−33570.211	−134790.028	23.900	6.058
MCT 10‐12‐111‐110	−16902.548	−84307.025	−33570.211	−134792.122	24.200	8.166

### 2.2 Interface Microstructure Evolution of Mg/Cu/Ta Sheets

**Figure 3:**
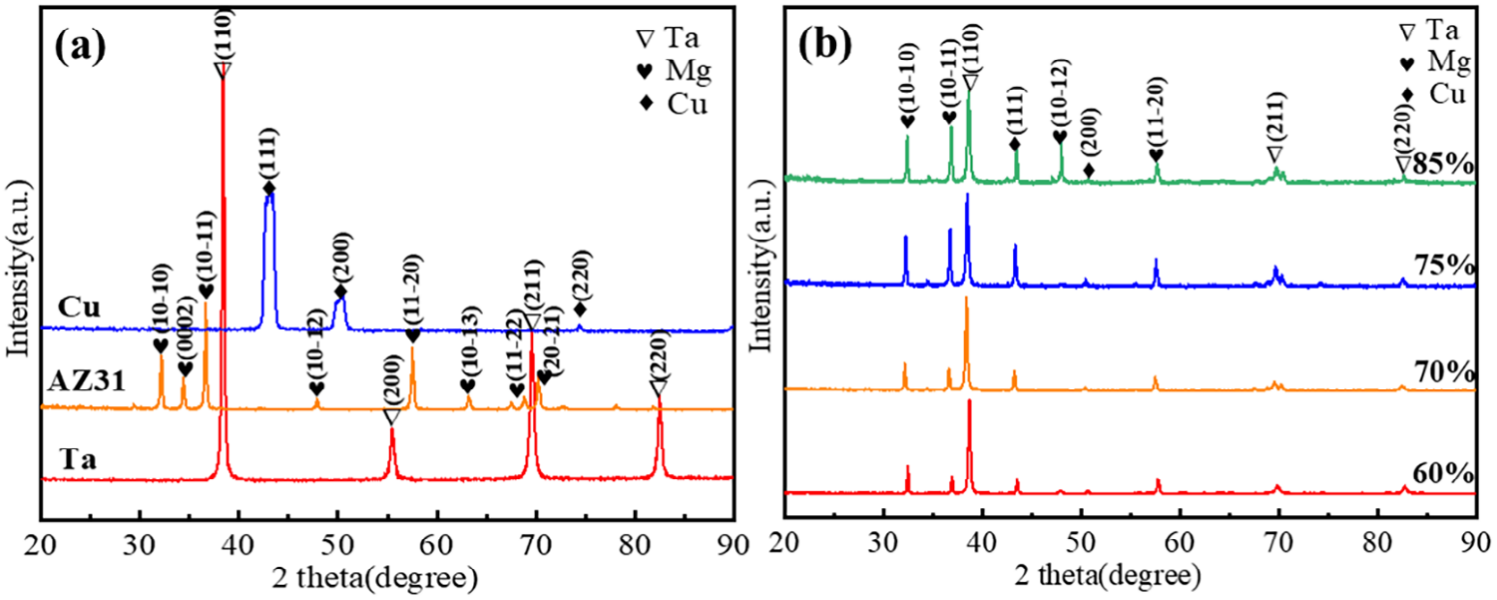
XRD diffraction patterns obtained for raw sheets and rolled laminated sheets: a) raw sheets, and b) rolled laminated sheets with different reductions (60%, 70%, 75%, 85%).

**Figure** **3** shows the XRD patterns of the raw materials and prepared Mg/Cu/Ta (MCT) laminated sheets (rolling direction × normal direction surfaces). It can be seen that the severe deformation (rolling process) optimized the appropriate crystalline orientation: the main peaks of Mg alloy (*hcp*) were prismatic (10‐10)_Mg_ plane, pyramidal (10‐11)_Mg_, and (10‐12)_Mg_ plane. The basal (0002)_Mg_ plane has a relatively low intensity due to most of these planes paralleling the RD direction. For Cu (*fcc*) and Ta (*bcc*) layers, the (111)_Cu_ and (110)_Ta_ planes are predominant, which came from the original Ta matrix containing other (200)_Ta_ and (211)_Ta_ planes, too. These theoretical interface designs are consistent with the experimental crystal plane distributions observed in the raw materials and rolling laminated sheets, where the dominant slip planes of Mg, Cu, and Ta layers correspond to the selected interfacial configurations.

**Figure 4:**
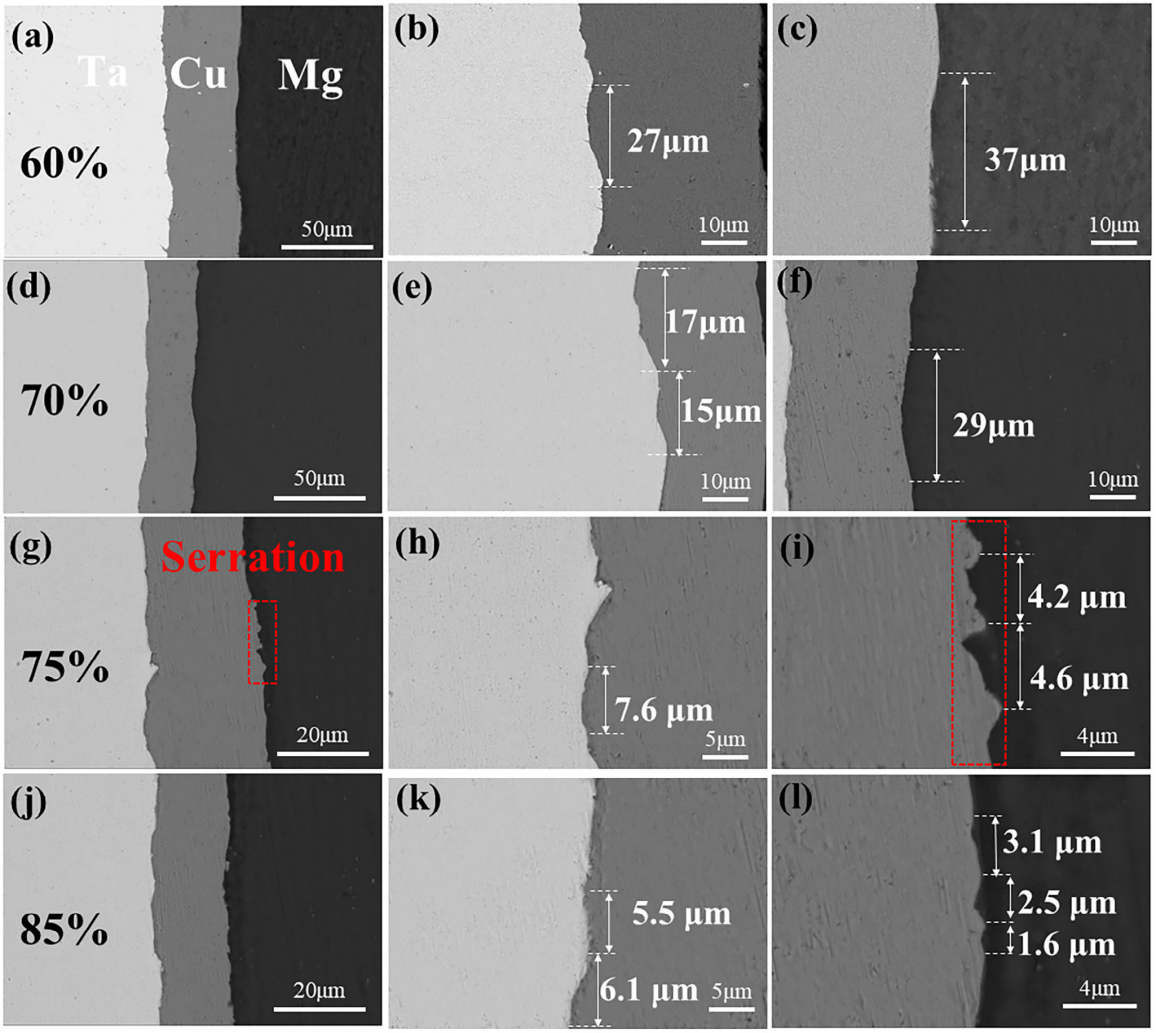
SEM morphology of Ta/Cu and Cu/Mg interfaces of laminated sheets obtained with different rolling reductions: a–c) 60%, d–f) 70%, g–i) 75%, and j–l) 85%.

**Figure** **4** displays the interfacial microstructure evolution of Mg/Cu/Ta (MCT) rolled sheets under different thickness reduction ratios (60–80%) through scanning electron microscopy (SEM) analysis. The interfacial zones maintained a structural continuity with curvilinear configurations, which comprised two distinct regions: Mg/Cu and Cu/Ta interface regions. Cu/Mg interface shows relatively planar morphology, while the Ta/Cu interface exhibits the characteristic with the serrated shapes, and the serration sizes are varying along both lateral and longitudinal directions. At 60% reduction (Figure 4a–c), shallow‐depth serrations (∼ 2 µm penetration) dominated both interfaces, measuring 27 ± 5 µm (Cu/Ta) and 37 ± 4 µm (Mg/Cu) along the RD direction (serration length). With deformation to 70% reduction, induced deeper serration penetration (∼ 5 µm) with reduced serration length: Cu/Ta:15 ± 5 µm; Mg/Cu:29 ± 5 µm. Further increasing reduction to 75–85% enhanced interface refinement, generating high‐density nanoscale serrations with spacing reduced to 6.6 ± 1 µm (Cu/Ta) and 3.1 ± 1.5 µm (Mg/Cu). This intensified interface curvature geometry effectively amplifies the actual contact area between Ta/Cu and Cu/Mg atoms by facilitating mechanical interlocking effects.

**Figure 5:**
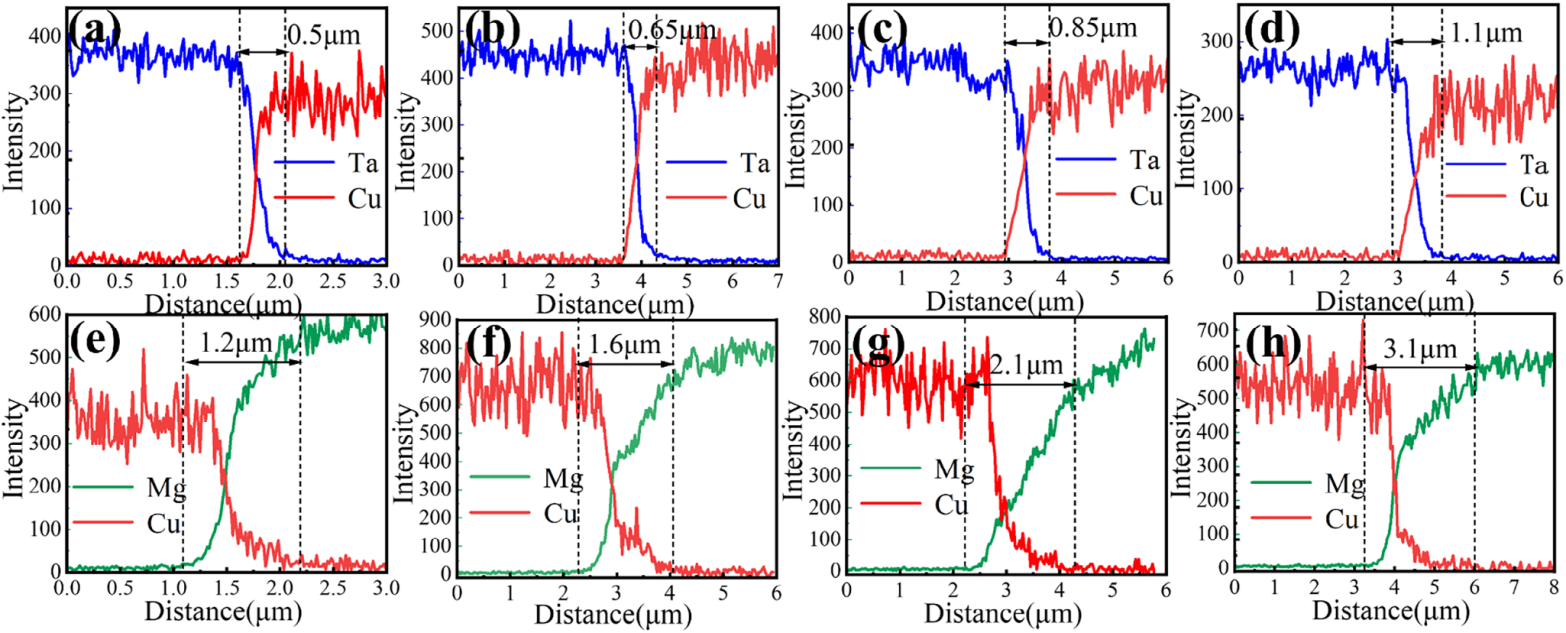
EDS line scanning across Ta/Cu (a–d) and Cu/Mg (e–h) interfaces of laminated sheets obtained with different rolling reductions: a,e) 60%, b,f) 70%, c,g) 75%, and d,h) 85%.

The atomic interdiffusion around these interfacial regions was quantitatively characterized by energy dispersive spectroscopy (EDS), as shown in **Figure** **5**. The analysis revealed significant compositional gradients at the Mg/Cu interface, where the diffusion zone width exhibited a thickness reduction‐dependent expansion from 1.2 ± 0.3 µm to 3.1 ± 0.4 µm under increasing deformation (60–85% reduction). In contrast, the Cu/Ta interface displayed restricted interdiffusion with a narrow diffusion zone of 0.5–1.1 µm. This distinct diffusion asymmetry highlights deformation‐driven interfacial activity differences: the Mg/Cu system follows conventional thermal diffusion kinetics, and the observed diffusion profiles confirm the establishment of metallurgical bonding at both interfaces regions during the rolling process.

**Figure 6:**
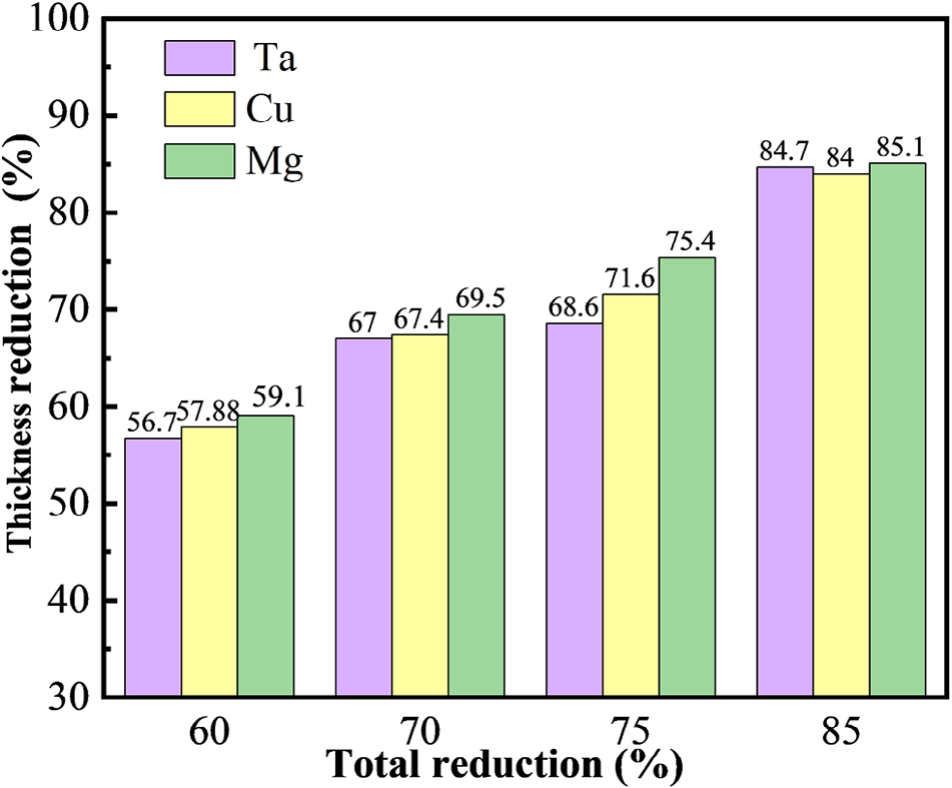
The thickness and reduction of Ta, Cu, and Mg layers after rolling.

**Figure** **6** quantifies the thickness evolution of individual metallic layer (Ta, Cu, Mg) with increasing rolling reductions. All constituent layer demonstrated non‐uniform plastic deformation that intensified with increasing rolling reduction. Notably, the Mg layer exhibited slightly greater thickness reduction compared to both Ta and Cu layers across all processing conditions. The intermediate Cu foil displayed moderate deformation characteristics, with its plastic flow behavior proving critical for maintaining interfacial coherence during the MCT sheet forming process.

**Figure 7:**
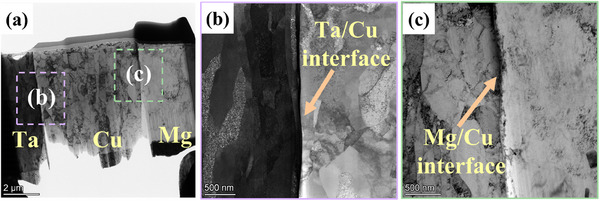
TEM morphology of the Mg/Cu/Ta interfaces, a) Ta /Cu /Mg (MCT) interface, b) Ta/Cu interface, and c) Cu/Mg interface.

**Figure** **7** shows the transmission electron microscope (TEM) morphology of the MCT interface zones. The low‐magnification bright‐field image demonstrates structurally continuous interface zones encompassing Mg, Cu, and Ta layer, as shown in Figure 7a. Figure 7b,c displays the atomic Mg/Cu and Cu/Ta interfaces, no intermetallic compounds are observed around these interfaces.

**Figure 8:**
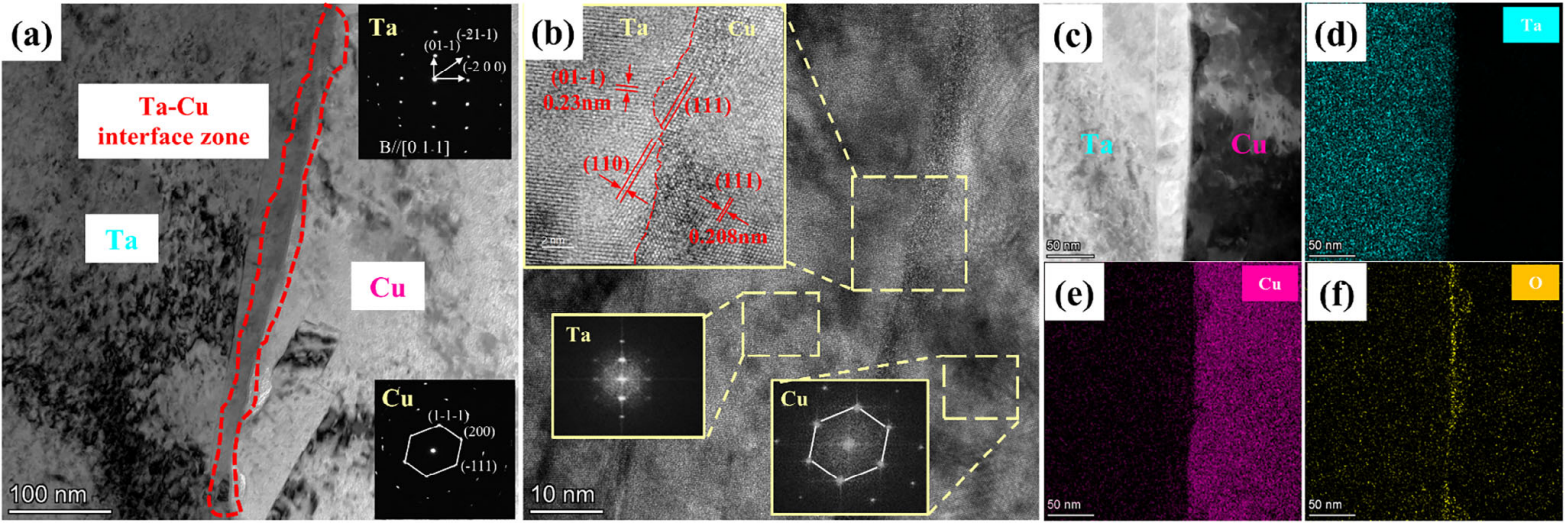
TEM morphology of the Ta/Cu interfaces, a) Ta/Cu interfaces and the selected area electron diffraction patterns (SAED) of Ta and Cu layer around the interface, b) high‐resolution transmission electron microscopy (HRTEM) image of the Ta/Cu interface, c) high‐angle annular dark‐field (HAADF) morphology of the Ta/Cu interface and d–f) EDS mapping.

The Ta/Cu interface zone is shown in **Figure** **8**. A high density of dislocations is observed around the Ta/Cu layers. The Ta/Cu interface exhibits a semi‐coherent lattice relationship, {110}_Ta_ // {111}_Cu_, and their interplane spacing are similar: d({111}_Cu_) = 0.21 nm and d({110}_Ta_) = 0.23 nm, as shown in Figure 8b. There are significant atoms diffused into each other, the atomic diffusion intensity gradually decreases from the matrix (Figure 8c–f). A small amount of O was also detected, mainly due to slight oxidation on the surface during heat treatment.

**Figure 9:**
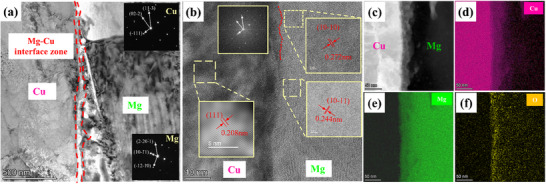
TEM morphology of the Cu/Mg interfaces, a) Cu/Mg interfaces and the SAED patterns of Cu and Mg around the interface, b) HRTEM image of the Cu/Mg interface, c) HAADF morphology of the Cu/Mg interface and d–f) EDS mapping.

**Figure** **9** shows the TEM images of the Mg/Cu interface zone. The Mg/Cu interface exhibited a significant atom interdiffusion zone, indicating a robust metallurgical bonding, compared to that of the Cu/Ta interface. Figure 9b exhibits the HRTEM image around the interface, revealing that Cu and Mg layers kept a semi‐coherent crystallographic relationship of {10‐10}_Mg_ // {111}_Cu_.

### 2.3 Mechanical Behaviors of Mg/Cu/Ta Sheets

**Figure 10:**
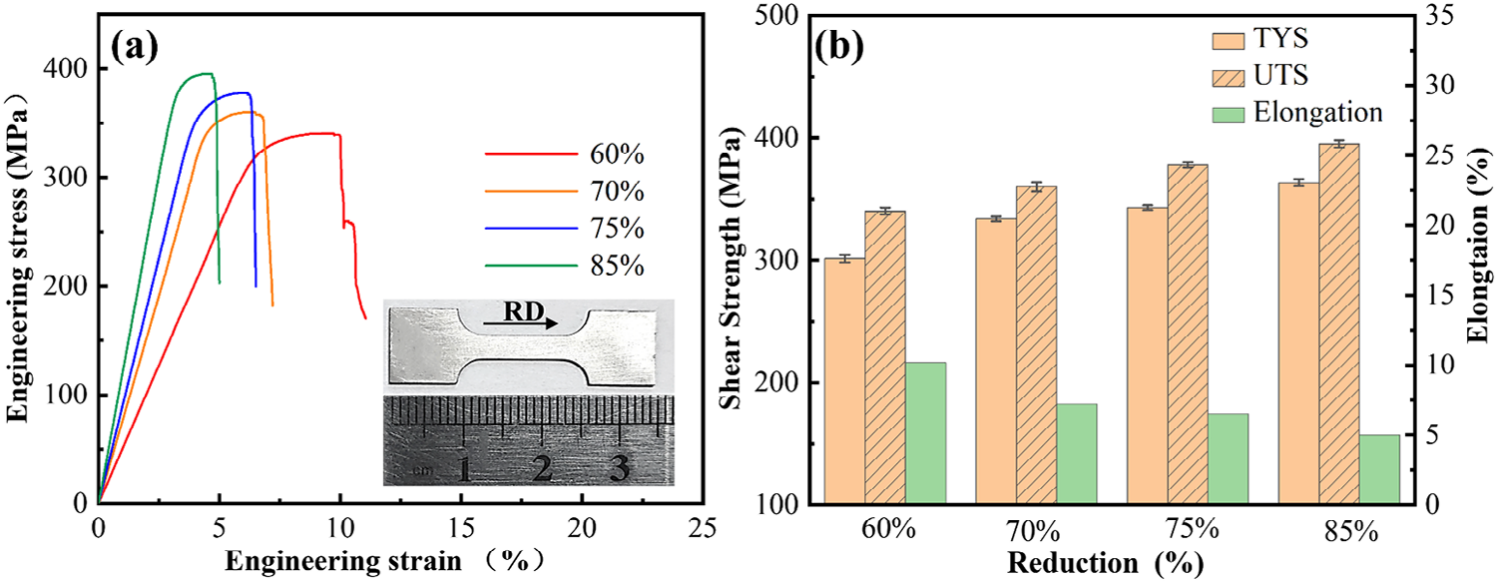
The tensile mechanical behaviors of Mg/Cu/Ta laminated sheets for different rolling reductions, a) the stress–strain curves, and b) the TYS, UTS values of laminated sheets, as well as EL.

**Figure** **10**a presents the stress‐strain curves of MCT sheets under varying rolling reduction conditions. The MCT laminated sheets exhibit notable strength‐plasticity synergy. As the rolling reduction increased from 60% to 85%, both ultimate tensile strength (UTS) and tensile yield strength (TYS) gradually increased, with TYS rising from 301 to 363 MPa and UTS increasing from 340 to 395 MPa. Concurrently, the elongation (EL) displayed a slight reduction from 10.2% to 5%, correlating with a moderate decline in overall plasticity as depicted in Figure 10b.

**Figure 11:**
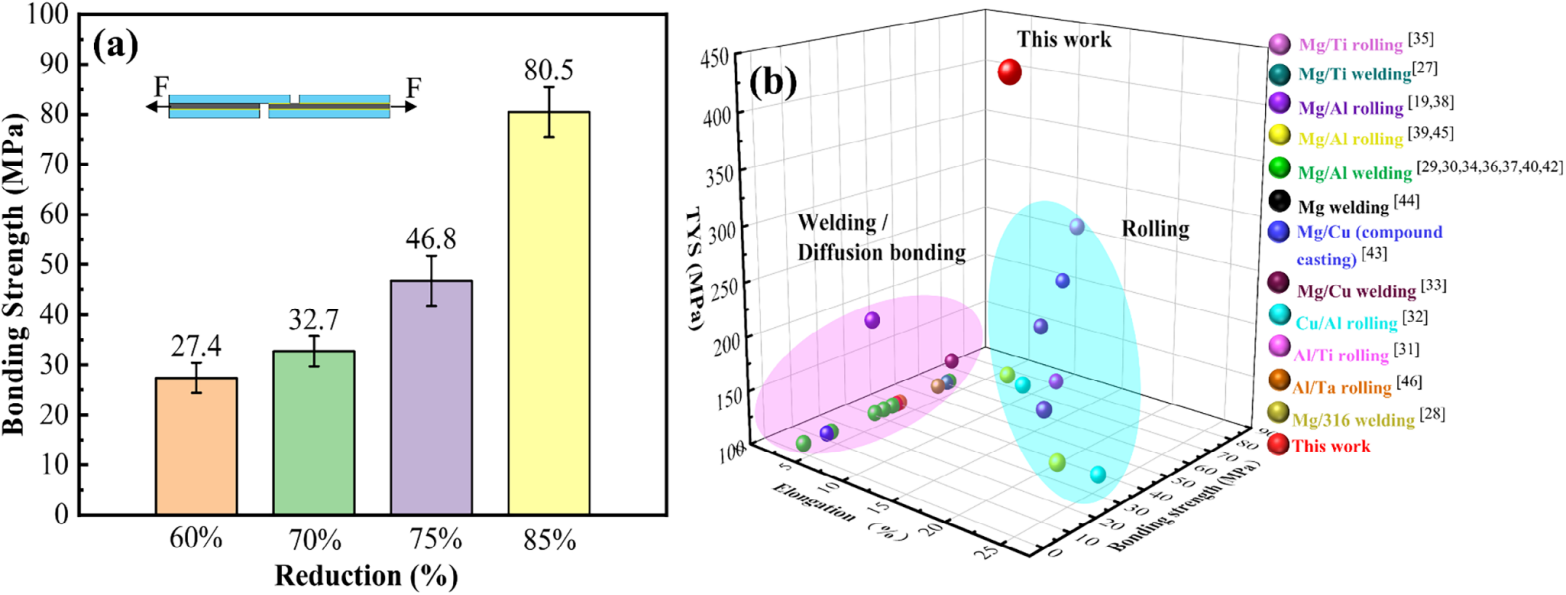
Interface bonding mechanical properties of MCT sheets and those reported Mg‐based LMCs, a) the interface bonding strength of MCT laminated sheets for different rolling reductions, and b) the tensile comprehensive mechanical properties of those reported Mg‐based LMCs.^[^ ^27^, ^28^, ^29^, ^30^, ^31^, ^32^, ^33^, ^34^, ^35^, ^36^, ^37^, ^38^, ^39^, ^40^, ^41^, ^42^, ^43^, ^44^, ^45^, ^46^ ^]^

The interface shear test was systematically performed to quantitatively evaluate interfacial bond behavior under different processing conditions, as shown in **Figure** **11**a. The bonding strength of MCT sheets demonstrates strong thickness reduction dependence. At 60–70% thickness reduction (or initial processing stages), the interface exhibits comparatively low bond strength (27.4–32.7 MPa). Crucially, a threshold phenomenon emerges at 75% reduction, where interfacial integrity undergoes dramatic enhancement, increased by a 71% strength improvement to 46.8 MPa. This transition correlates with intensified shear deformation promoting effective atomic interdiffusion. The synergistic effect culminates at an 85% reduction, attaining a peak bond strength of 80.5 MPa, which is about a 194% increase over the initial stage, verifying that progressive strain critically modifies interfacial characteristics.

These mechanical advancements position MCT sheets advantageously within the Mg‐based LMC landscape. As benchmarked against established Mg‐based alloys in Figure 11b, ^[^ ^12^, ^14^, ^18^ ^]^ the present material achieves unprecedented 22–37% bond strength superiority over conventional welded/diffusion‐bonded counterparts while maintaining exceptional strength‐plasticity balance (UTS = 395 MPa, EL = 5%). Such performance divergence originates from the unique interfacial structure behavior and its bonding mechanism that simultaneously optimizes interfacial cohesion and matrix strengthening.

**Figure 12:**
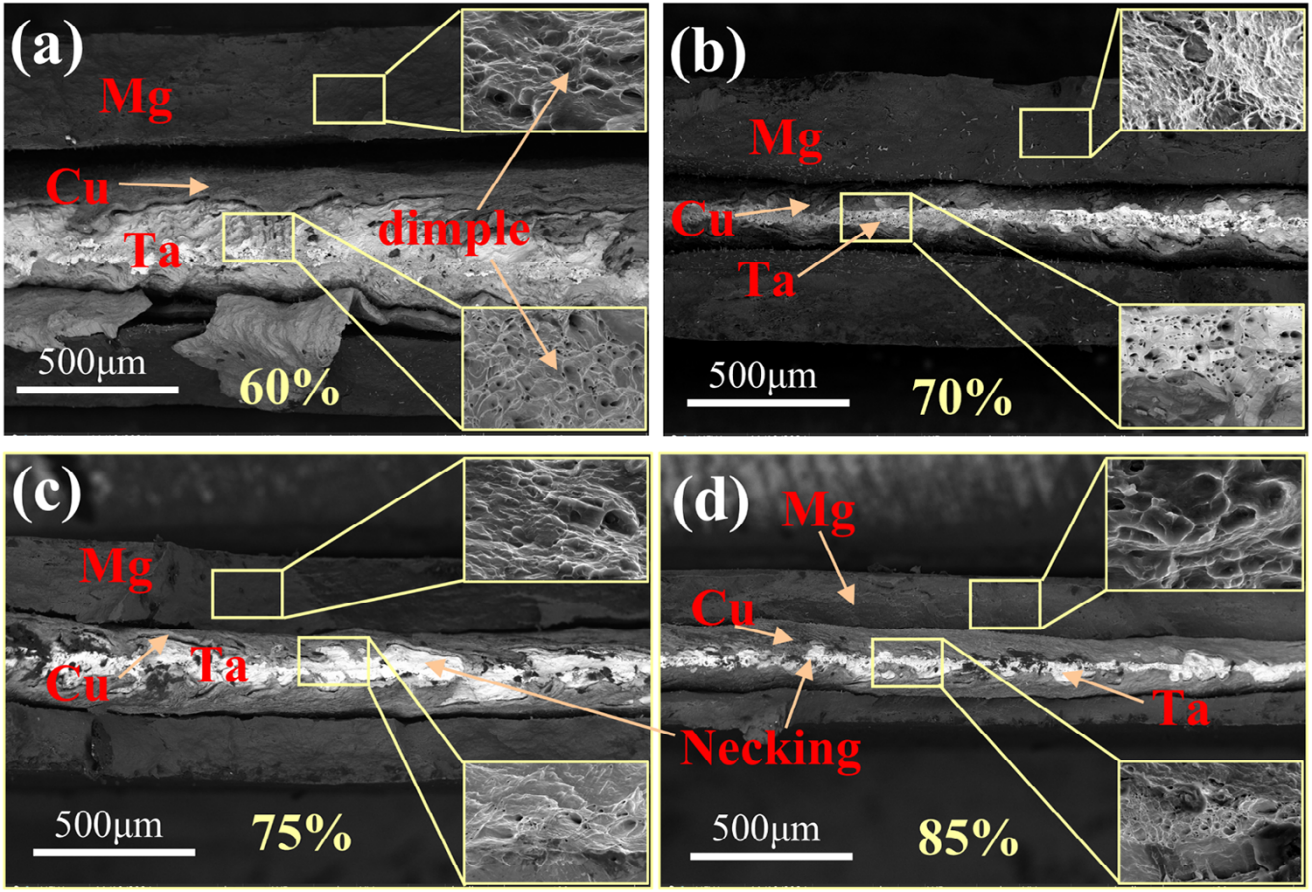
The fracture morphology of Mg/Cu/Ta laminated sheets for different rolling reductions: a) 60%, b) 70%, c) 75%, and d) 85%.

**Figure** **12** shows the tensile fracture morphology of MCT sheets, revealing distinct cracking in both Mg and Cu layers, while Cu/Ta interfacial demonstrate strong adhesion. At the lower reduction ratio of 60%, poor interlayer bonding is evident, with partial Cu film detachment from the Ta matrix (Figure 12a). As reduction increases, significant microstructural evolution occurs: the Ta layer exhibits pronounced necking, while both Mg and Ta layers develop dimpled fracture surfaces. Meanwhile, the distinct Poisson's ratios of Mg (0.29–0.35), Cu (∼0.33), and Ta (∼0.35) lead to significant disparities in transverse strain under uniaxial loading conditions.^[^ ^47^ ^]^ Specifically, those materials with higher Poisson's ratios‐particularly anisotropic metals like Ta and Cu, exhibiting greater lateral contraction. In contrast, Mg, with its comparably lower Poisson's ratio, demonstrates reduced contraction magnitude.

This morphological transition directly reflects the development of mechanically interlocked interfaces. Lower reductions (60–70%) produce insufficient interface serrations, resulting in weak bonding and interfacial decohesion (Cu/Ta delamination). In contrast, higher reductions (75–85%) generate refined interface serrations that enhance mechanical interlocking, diverting crack propagation from the interface into the metal matrix. The emergence of dimples in Mg/Ta layers combined with Ta layer necking confirms a failure mode transition from interfacial separation to matrix‐dominated ductile fracture, ultimately contributing to enhanced tensile strength.

**Figure 13:**
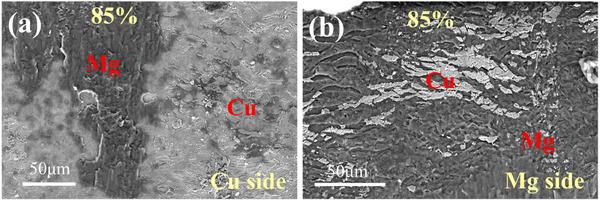
The shear fracture morphology of Mg/Cu/Ta laminated sheets with an 85% rolling reduction: a) Cu side morphology, and b) Mg side morphology.

**Figure** **13** shows the shear fracture morphologies of MCT, revealing distinct interfacial behavior between the constituent layers. Notably, the Cu/Ta interface maintains structural integrity despite failure at the Cu/Mg interface. Whereas the Cu/Mg interface exhibits failure associated with a larger interplanar spacing mismatch and lower interfacial separation work. Both fracture surfaces exhibit pronounced corrugated topography with uneven bulges, accompanied by residual Cu fragments firmly adhering to the Mg matrix. These morphological features demonstrate a mechanical interlocking mechanism where Cu undergoes plastic deformation and embeds into the Mg matrix during shear loading.

## 3 Discussion

### 3.1 Heterogeneous Interfacial Microstructure Characteristics for MCT Sheets

**Figure 14:**
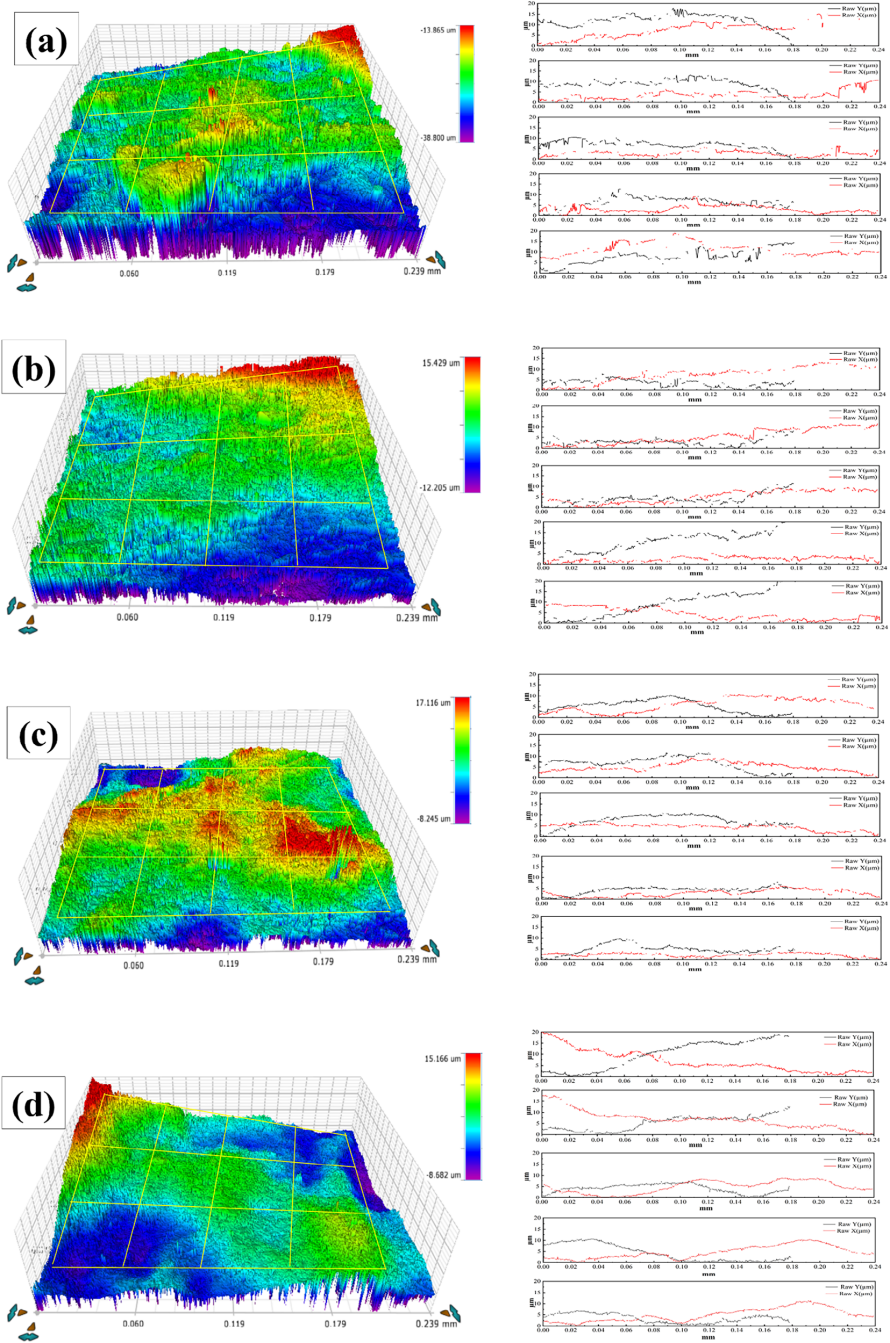
3D morphology scanning results of the Cu side of the peeling surface obtained with different rolling reductions, accompanied by the corresponding height distribution along the rolling direction: a) 60%, b) 70%, c) 75%, and d) 85%.

The MCT sheets exhibit exceptional bonding strength and superior tensile mechanical properties, attributed to their architecturally optimized interfacial microstructure with three distinctive characteristics. Primarily, the interfaces in situ form through structural design utilizing deformation disparity between constituent metals under uniform compressive loading during rolling, contrasting with conventional non‐uniform severe deformation approaches.^[^ ^19^, ^48^ ^]^ Secondarily, this multi‐phase configuration comprises: 1) Three metallic constituents with distinct crystal structures (*hcp*‐Mg, *fcc*‐Cu, *bcc*‐Ta); 2) Five stratified layers with coherent lattice matching; 3) Dual hetero‐interfaces: *hcp*/*fcc* (Mg/Cu) and *fcc*/*bcc* (Cu/Ta) interfacial architectures. Third, the clear atomic interdiffusion occurs around the two interfaces regions, the atomic interdiffusion zone depth of the Mg/Cu interface is more than 3 times that at the Cu/Ta interface, and the maximum diffusion width is ∼ 3.8 µm. The above MCT sheets possess in situ serrated interlocking interface features, which are typically only found in special large deformations, such as typical hard plate rolling ^[^ ^19^ ^]^, and rolling after processing trapezoidal interfaces on the plate surface.^[^ ^41^ ^]^ This special interface feature was formed without any special treatment in this study, indicating that it has a special formation mechanism. Besides, the interfacial topology exhibits pronounced undulations along rolling (RD) and transverse (TD) directions, featuring micrometer‐scale mechanical interlocking structures. As evidenced by 3D profilometry (**Figure** **14**), the Ta/Cu interface displays serration widths of 5.5–27 µm, while the Cu/Mg interface demonstrates greater morphological complexity with widths of 1.6–37 µm and interlocking depths of 6–15 µm.

Fractographic analysis reveals preferential failure at Cu/Mg interfaces, as shown in Figures 11 and 13, indicating the stronger bonding at Ta/Cu interfaces. Intriguingly, interfacial geometry shows an inverse correlation with bond strength: larger features (width: 15–27 µm; depth: 10–15 µm) correspond to lower strength (27.4–32.7 MPa), while optimized dimensions (width: 5.5–7.6 µm; depth: 6–10 µm) enable significant strengthening (46.8–80.5 MPa), surpassing conventional metallurgical bonding by 41‐63%. This dimensional dependence originates from stress concentration mitigation in refined interlocking structures, highlighting the critical role of strain‐controlled interface engineering.

**Figure 15:**
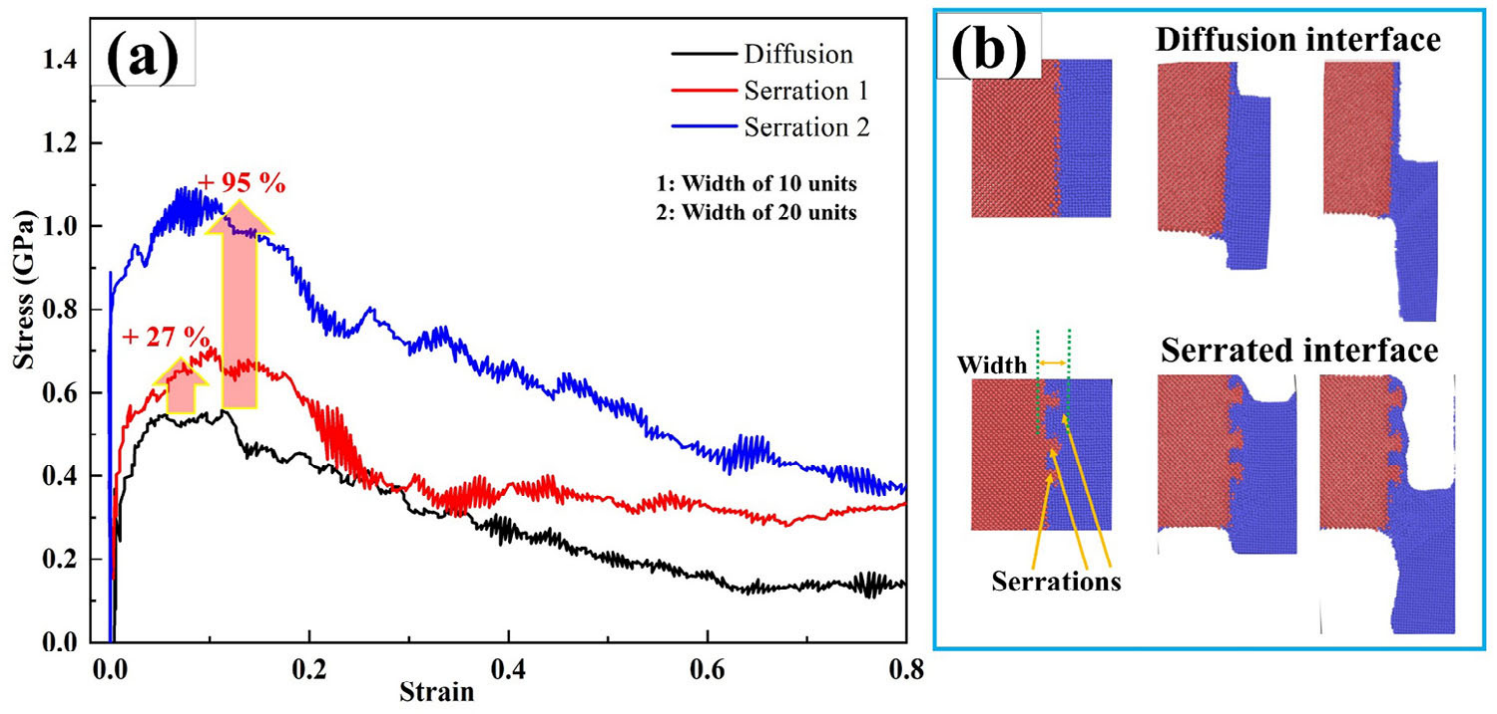
The tensile MD stress–strain behavior with different serration characters, a) the stress–strain curves, and b) MD models with different tensile processes.

The systematic investigation establishes that serrated interlocking morphology critically governs interfacial bonding strength, as shown in **Figure** **15**. The molecular dynamics simulations conclusively demonstrate a minimum 27% increase in interfacial bonding stress compared to non‐serrated configurations, with further stress enhancement reaching up to 95% through optimized serration width modulation. These findings unequivocally validate the efficacy of geometric interlocking for achieving superior interfacial adhesion. The two distinct interface configurations demonstrate a bond strength enhancement in serrated interfaces compared to planar diffusion‐bonded counterparts, this improvement originates from reduced stress concentrations in refined serration geometries, fundamentally altering load transfer mechanisms.

### 3.2 Interfaces (*hcp/fcc/bcc*) Forming Mechanism of MCT Sheets

**Figure 16:**
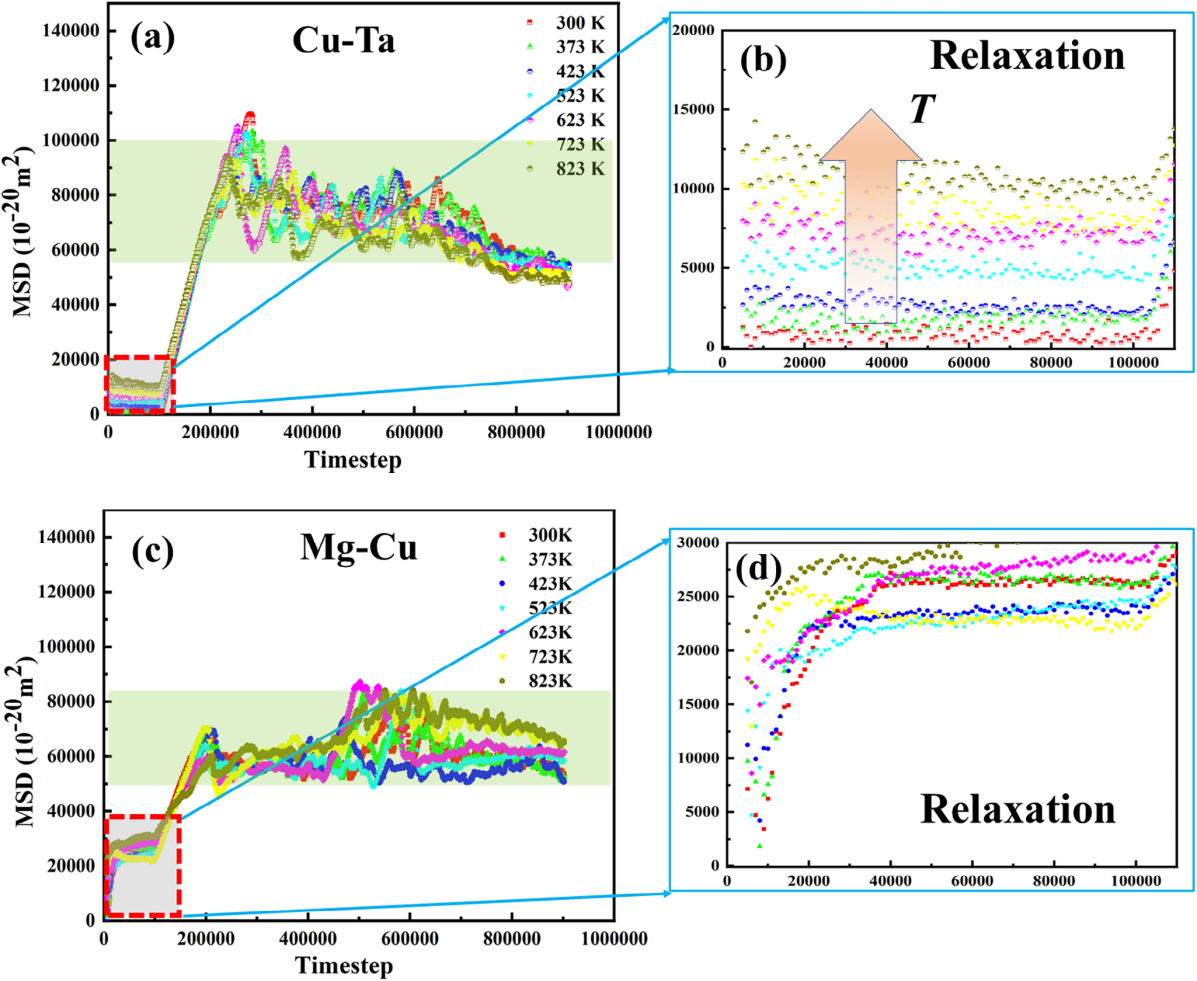
The MSD values of interfacial atoms with time at different temperatures: a,b) Ta/Cu, and c,d) Mg /Cu interface zone.

The formation mechanism of *hcp/fcc/bcc* interfaces in MCT sheets was investigated based on MD simulations using LAMMPS under thermo‐mechanical coupling conditions. As shown in **Figure** **16**, the temperature‐dependent mean square displacement (MSD) analysis reveals distinct atomic diffusion behaviors between Mg/Cu and Cu/Ta interfaces during rolling processes (300–800 K, or ∼25–527 °C). The Mg‐Cu interface exhibits inherently higher diffusion coefficients compared to the Cu‐Ta interface in this temperature range. Notably, the Cu‐Ta interface demonstrates greater thermal sensitivity, its interdiffusion increases more significantly with temperature than Mg‐Cu pairs, as evidenced by the steeper MSD slope in Figure 16b,d.

Additionally, plastic deformation induces a nonlinear interdiffusion response, characterized by an initial linear growth stage (ε = 0‐0.1) where dislocation activates to slip, followed by a high strain regime (ε>0.1) where dynamic recovery reduces diffusion efficiency. Crucially, the coupled thermal‐mechanical loading synergistically enhances interfacial atomic interdiffusion, producing coordinated MSD magnitudes (60 000–100 000 × 10^−20^m^2^) across all interface components during the large‐strain stage (ε>0.3). These results establish that interface evolution in MCT sheets arises from the competition between thermally activated diffusion kinetics and strain‐mediated microstructural evolution.

**Figure 17:**
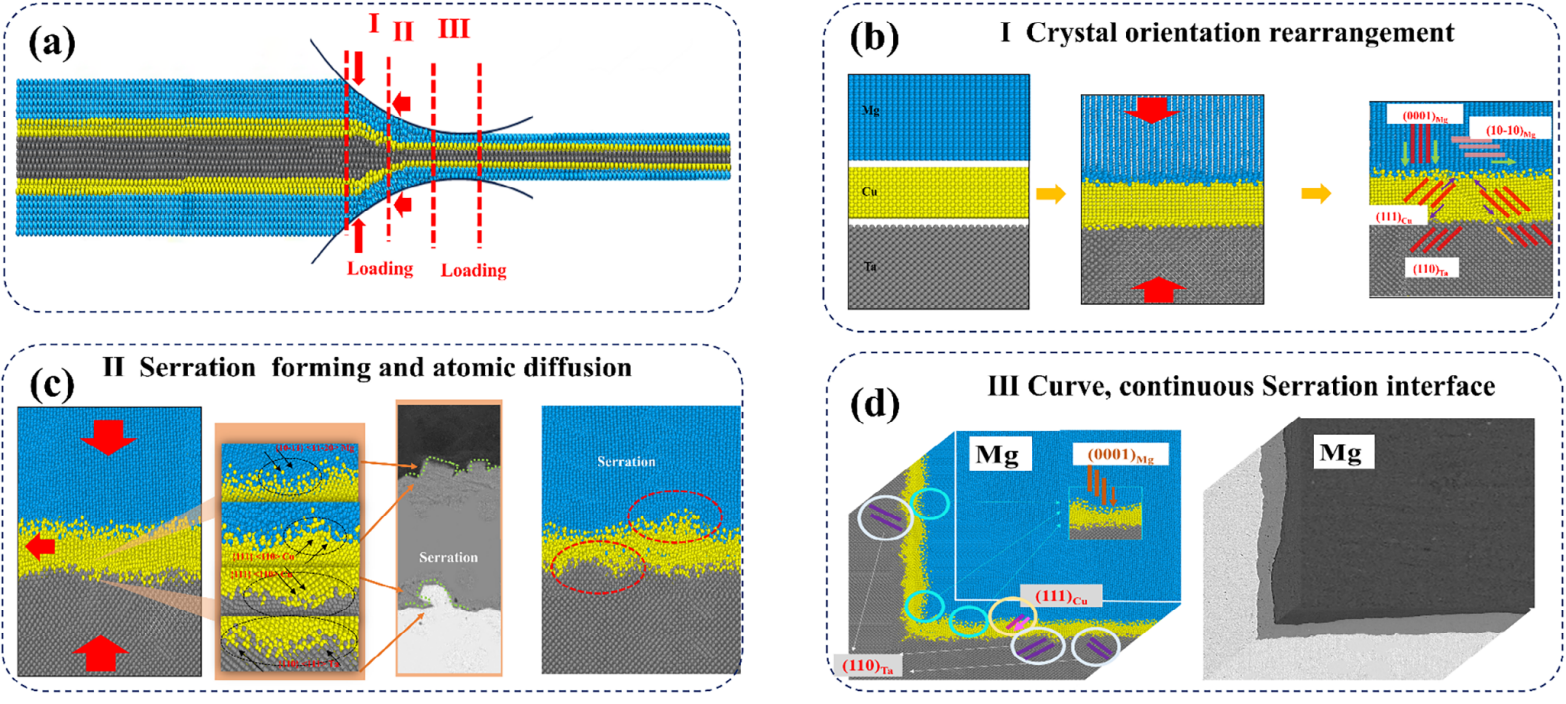
Schematics showing the hot rolling bonding mechanism for AZ31/Cu/Ta laminated composites.

This unique interface formation process comprises 3 distinct stages. (1) Crystallographic orientation optimization via dissimilar atomic contact (**Figure** **17**a,b). Microscopic protrusions and depressions on these surfaces come into contact, meanwhile, these metals occur plastic deformation with planes slipping to the optimized orientation relationship, e.g., (0001)_Mg_/(111)_Cu_/(110)_Ta_, and <10‐10>_Mg_˔ ⊥ <110>_Cu_. The accumulated deformation results in surface cracks, and then the fresh metal is squeezed and comes into contact with each other, forming an initial thermomechanical coupling interface. Besides, the crystal planes at the interface regions occur rearranging to form lower‐energy textures such as (0001)_Mg_/ (111)_Cu_/ (110)_Ta_ and (10‐10)_Mg_/ (111)_Cu_/ (110)_Ta_.

(2) Strain‐driven micro‐sawtooth generation through dislocation slip competition (Figure 17c). As the hot‐rolling process progresses, both temperature and pressure enhance atomic interdiffusion. On the one hand, atoms diffuse laterally through lattice defects (interface extension) and penetrate each other (interlocking serrations), leading to gradual changes in the interface composition and then forming the atomic diffusion layer. On the other hand, significant strain induces Mg, Cu, and Ta to undergo extensive plastic deformation and mutual penetration into each other's matrix. Specifically, Mg prismatic planes dislocation slipping became the leading dislocation and entered into the Cu layer side, due to the semi‐coherent relation of each other's closed stacking planes. Meantime, Cu shows the obviously {111}<110>_Cu_ slipping enter into the Ta layer side, too, as well as {110}<111>_Ta_ entering into the Cu layer. These leading dislocations were hindered by the counterpart side, and these slipping planes formed with the convex shape, and then gradually formed a serration around the interface.

(3) Geometric interlocking completion via interface self‐assembly (Figure 17d). With the plasticity deformation increasing, the RD partial force along with the increase of strain, the serration number increases at both interface zones, Mg/Cu interface and Cu/Ta interface. Along with local microstructural inhomogeneities caused by micro‐point defects, plane slipping, and dislocation pile‐ups, results in the formation of continuous mechanical interlocking interfaces with a depth (along the interface normal, 6–10 µm) and width (along the RD direction, Ta/Cu: 5.5 ± 2 µm, Cu/Mg: 1.6 ± 1 µm).

## 4 Conclusion

Interface engineering of heterogeneous laminated metal composites (LMCs) establishes fundamental guidelines for developing advanced structural materials. This work focuses on the design and formation mechanisms of hetero‐crystalline interfaces with containing different crystalline lattice (*hcp/fcc/bcc*) with optimized strength‐ductility synergy. The interface zone's structure and mechanical behavior evolution of Mg/Cu/Ta samples prepared is systematically investigated. The main findings are shown below:

1. DFT calculations identify optimal crystallographic matching between Mg and Cu, Ta counterparts ((111)_Cu_ and (110)_Ta_). While (10‐12)_Mg_/(111)_Cu_/(110)_Ta_ configuration exhibits minimal lattice mismatch (δ < 25%), and (0001)_Mg_/(111)_Cu_/(110)_Ta_ shows enhanced thermal stability interfacial separation work of 34.947 J m^−2^. And then the MCT LMC sheets containing (0001)_Mg_/(111)_Cu_/(110)_Ta_ configuration interface was successfully prepared, it shows excellent mechanical behaviors: the interface bonding strength reached up to 80.5 MPa, and the tensile strengths were ranging from 340 to 395 MPa and elongation of 5–10.2%.
2. The resultant interfaces feature depth‐modulated serrations (6–15 µm) with multiscale atomic interdiffusion (Mg‐Cu diffusion depth: 3.8 µm vs Cu‐Ta: 1.2 µm), forming 5‐layer transitional zones containing dual interfaces (*hcp/fcc* Mg/Cu and *fcc/bcc* Cu/Ta). Crucially, these hierarchically structured interfaces develop through intrinsic deformation mechanisms rather than external geometric patterning.
3. Microstructural characterization and MD simulation reveal in situ formation of self‐organized *hcp/fcc/bcc* interfaces through three evolutionary stages: (1) crystallographic orientation optimization via dissimilar atomic contact, (2) strain‐driven micro‐sawtooth generation through dislocation slip competition, and (3) geometric interlocking completion via interface self‐assembly.

## 5 Experimental Section

### Materials and Simulation

**Figure 18:**
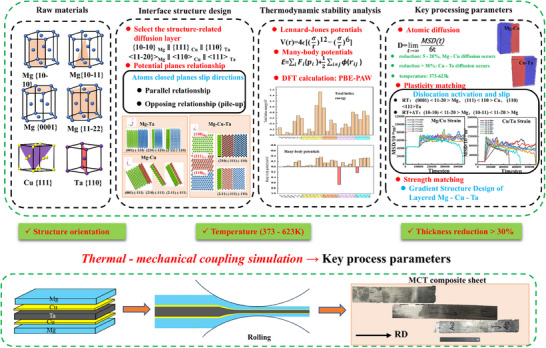
The schematic diagram of the design dissimilar lattice interfaces with strength/plasticity synergistic deformation.

Magnesium alloy (AZ31, 100 mm × 50 mm × 1 mm), Cu foils (100 mm × 50 mm × 0.1 mm), and pure Ta sheets (100 mm × 50 mm × 1 mm) were employed as *hcp*, *fcc*, and *bcc* metals, respectively. **Figure** **18** shows the schematic diagram of this study from designing interfaces to preparing LMC material. First, the multiple layers *hcp/fcc/bcc* interface models with different orientation relationships were built, and the thermodynamic stability and lattice mismatch were analyzed by MD simulation and DFT calculation. Subsequently, the effect of key factors (temperature, reduction rate, and strain) on the atomic interface formation was simulated, and their value range was determined. Finally, adopting the hot rolling method and optimizing the processing parameters to prepare the Mg/Cu/Ta (MCT) LMC materials.

Materials Studio (MS) with Cambridge Serial Total Energy Package (CASTEP) was used to calculate the dissimilar interfaces energy. The exchange‐correlation energy was evaluated using the Perdew–Burke–Ernzerhof (PBE) functional within the generalized gradient approximation (GGA) framework. During the optimization process, the ultra‐soft pseudopotential relaxed the conventional conditions and was used as a plane‐wave basis set for calculation through the self‐consistent iterative method. Electronic relaxation was handled by the Pulay density mixing scheme and the Broyden‐Fletcher‐Goldfarb‐Shanno (BFGS) conjugate gradient method. To ensure computational efficiency and achieve the accuracy of the system energy set, the energy cutoff was 350 eV based on the results of the convergence test. SCF tolerance set to ultrafine, the k‐point grid for Brillouin zone sampling was determined using the Monkhorst‐Pack scheme, 3 × 8, and the total energy was converged to within 1.0 × 10^−5^ eV atom^−^. Molecule dynamics (MD) simulation was conducted by the Large‐scale Atomic/Molecular Massive Parallel Simulator (LAMMPS), and the modified embedded atom method (MEAM) was adopted. The time step was 1 fs, and the simulation box size was 100 × 50 × 100 units. The initial model was fully relaxed for 20–50 *ps* with the constant‐pressure and constant‐temperature (NPT) ensemble, the system temperature was controlled using a Nose‐Hoover thermostat, ranging from 25 to 550 °C. The strain rate was calculated using Equation (3).^[^ ^18^ ^]^

(3) $$\varepsilon =2\times V\times \sqrt{\frac{H-h}{R}}\times \frac{1}{H+h}$$

where Ɛ is the mean strain rate, *V* is the linear velocity, *H* and *h* mean the rolled sheet's thickness before and after rolling, and *R* indicates the radius of a rolling ball. And then, the plastic strain rate was ≈0.008 s^−1^. Open‐source software OVITO was used for the visualization and analysis of point defects (Wigner‐Seitz defect method).

Before the rolling process, the AZ31 sheet was annealed at 350 °C for 1 h. Similarly, the raw Ta sheet was subjected to annealing treatments at 1200 °C for 2 h, and the Cu film was annealed at 500 °C for 1 h. The surfaces of all the sheets were brushed with a steel brush and subsequently degreased using ethyl alcohol. Then, the materials were stacked in the sequence of AZ31/Cu/Ta/Cu/AZ31 and fixed with rivets. The total thickness of the initial composites was ∼ 3.2 mm. The stacked sheets were then rolled at a temperature of 400 °C, with a rolling speed of 22 mm s^−1^. The total reduction rate was ∼60%, 70%, 75%, and 85%, respectively.

### Microstructure Characterization

The samples for microstructure investigation were obtained from the RD × ND surfaces of the rolled sheets. Scanning electron microscopy (SEM, Thermo‐Scientific Quattro) with energy dispersive spectroscopy (EDS) and transmission electron microscopy (TEM, Thermo Scientific Talos F200X G2) were applied to interface morphology. 3D Optical Profilometer (Contour GT‐K‐ELITE) was used to characterize the deformed sheets' contour. X‐ray diffraction (XRD, Haoyuan DX‐2700BH) was also used to analyze the phases of the sheets before/after rolling.

### Mechanical Testing

The tensile and interface bonding mechanical tests were performed using an electronic universal testing machine (WGW‐50) at room temperature (∼25 µC) with a strain‐rate of 1 × 10^−3^s^−1^, and each sample was tested three tests. The tensile samples were performed parallel to the rolling direction of the samples (Total specimen length: 34 mm, width of the gauge section: 3 mm, gauge length: 10 mm). The bonding strength *τ* is calculated using the formula *τ* = F/A, where F represents the shear force, and A represents the contact area between the shear slots of the sample.

## Conflict of Interest

The authors declare no conflict of interest.

## Author Contributions

W.L. contributed equally to this work. W.B.L. and X.Z.H. were responsible for the conceptualization and supervision of the study. Data curation and investigation were carried out by W.B.L., Z.L.Y., and H.Y.Z. Methodology was developed by Z.L.Y., W.B.L., X.Z.H., W.S.Z., and H.Y.Z. Funding acquisition and project administration were led by W.B.L., X.Z.H., and Z.Y.X. Resources were provided by H.Y.Z. and X.M.C. Visualization was performed by R.Q.X. and W.X. The original draft of the manuscript was written by Z.L.Y. and W.B.L. Writing, review, and editing were performed by W.B.L., Z.L.Y., W.X., R.Q.X., Z.Y.X., and X.M.C.

## Data Availability

The data that support the findings of this study are available from the corresponding author upon reasonable request.
